# Molecular Characteristics and Metastasis Predictor Genes of Triple-Negative Breast Cancer: A Clinical Study of Triple-Negative Breast Carcinomas

**DOI:** 10.1371/journal.pone.0045831

**Published:** 2012-09-25

**Authors:** Wen-Hung Kuo, Yao-Yin Chang, Liang-Chuan Lai, Mong-Hsun Tsai, Chuhsing Kate Hsiao, King-Jen Chang, Eric Y. Chuang

**Affiliations:** 1 Graduate Institute of Biomedical Electronics and Bioinformatics, Department of Electrical Engineering, National Taiwan University, Taipei, Taiwan; 2 Department of Surgery, College of Medicine, National Taiwan University, Taipei, Taiwan; 3 Bioinformatics and Biostatistics Core, NTU Center of Genomic Medicine, Taipei, Taiwan; 4 Department of Physiology, College of Medicine, National Taiwan University, Taipei, Taiwan; 5 Institute of Biotechnology, College of Bio-resources and Agriculture, National Taiwan University, Taipei, Taiwan; 6 Department of Public Health, College of Public Health, National Taiwan University, Taipei, Taiwan; 7 Department of Surgery, Cheng Ching General Hospital, Taichung, Taiwan; University of Texas MD Anderson Cancer Center, United States of America

## Abstract

**Background:**

Triple-negative breast cancer is a subtype of breast cancer with aggressive tumor behavior and distinct disease etiology. Due to the lack of an effective targeted medicine, treatment options for triple-negative breast cancer are few and recurrence rates are high. Although various multi-gene prognostic markers have been proposed for the prediction of breast cancer outcome, most of them were proven clinically useful only for estrogen receptor-positive breast cancers. Reliable identification of triple-negative patients with a favorable prognosis is not yet possible.

**Methodology/Principal Findings:**

Clinicopathological information and microarray data from 157 invasive breast carcinomas were collected at National Taiwan University Hospital from 1995 to 2008. Gene expression data of 51 triple-negative and 106 luminal breast cancers were generated by oligonucleotide microarrays. Hierarchical clustering analysis revealed that the majority (94%) of triple-negative breast cancers were tightly clustered together carrying strong basal-like characteristics. A 45-gene prognostic signature giving 98% predictive accuracy in distant recurrence of our triple-negative patients was determined using the receiver operating characteristic analysis and leave-one-out cross validation. External validation of the prognostic signature in an independent microarray dataset of 59 early-stage triple-negative patients also obtained statistical significance (hazard ratio 2.29, 95% confidence interval (CI) 1.04–5.06, Cox *P* = 0.04), outperforming five other published breast cancer prognostic signatures. The 45-gene signature identified in this study revealed that TGF-β signaling of immune/inflammatory regulation may play an important role in distant metastatic invasion of triple-negative breast cancer.

**Conclusions/Significance:**

Gene expression data and recurrence information of triple-negative breast cancer were collected and analyzed in this study. A novel set of 45-gene signature was found to be statistically predictive in disease recurrence of triple-negative breast cancer. The 45-gene signature, if further validated, may be a clinically useful tool in risk assessment of distant recurrence for early-stage triple-negative patients.

## Introduction

Triple-negative breast cancer, immunohistochemically defined by lack of expression of estrogen receptor (ER), progesterone receptor (PR), and human epidermal growth factor receptor 2 (HER2), is an important phenotype of breast cancer that accounts for approximately 10–15% of all breast cancers [1]–[3]. The absence of ER, PR, and HER2 protein receptors on triple-negative breast tumors precludes many therapeutic options that incorporate hormonal drugs or monoclonal antibodies. As a result, triple-negative breast cancer is often associated with a worse prognosis and higher death rate than other subtypes of breast cancers [4]–[6].

The term “triple-negative breast cancer” is often used interchangeably with “basal-like breast cancer” due to strong histological similarities between the two breast cancer subtypes [7]–[10]. Basal-like breast tumors are preferentially low in ER and HER2 expression, and are significantly associated with several basal cytokeratin (CK) markers, including CK 5/6, CK 14, CK 17, and epidermal growth factor receptor (EGFR) [11]. Gene expression profiling pioneered by Perou *et al.* and Sorlie *et al.* showed that breast cancer can be reliably reclassified into five major subtypes (luminal A, luminal B, HER2/neu, basal-like, and normal breast-like) based on gene expression patterns from the intrinsic gene set [12], [13]. In their hierarchical clustering analyses, basal-like breast tumors were grouped together within a tight cluster showing high expression of basal cytokeratin genes (*KRT5* and *KRT17*) and low expression of luminal estrogen receptor gene *(ESR1*).

Although triple-negative breast cancer possesses many basal-like characteristics, equating triple-negative breast cancer with basal-like breast cancer is not fully supported by many studies [11], [14]–[17]. In an investigation of the association between triple-negative phenotype and basal cytokeratin markers, Tan *et al.* reported that 6 out of 31 (19.4%) triple-negative breast tumors were negative for basal makers (CK 5/6, CK 14, CK 17, and EGFR), while 15 out of 207 (6.3%) non-triple-negative tumors expressed basal makers [11]. An immunohistochemical validation of basal-like breast cancer by Nielsen *et al.* showed that the microarray-defined basal-like breast cancer could be effectively identified using a panel of four immunohistochemical markers (ER-, HER2-, CK 5/6+ and HER1+) with 100% specificity and 76% sensitivity [9].

**Figure 1:**
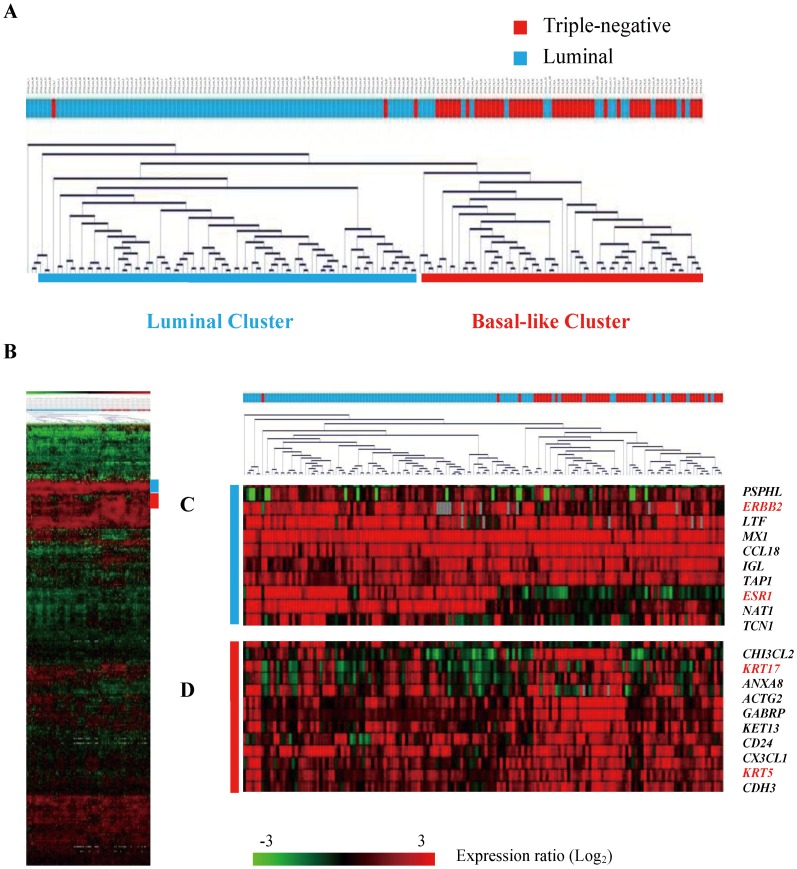
Hierarchical clustering analysis of 157 breast tumors (51 triple-negative and 106 luminal) using 261 intrinsic genes. The breast tumors were classified into two dominant clusters based on similarities in expression patterns. (A) The dendrogram depicts similarities in gene expression patterns of the breast tumors divided into two dominant clusters. Triple-negative tumors were colored red, and luminal tumors were colored blue. (B) Gene expression data from 261 intrinsic genes. Each row represents a gene and each column represents a breast tumor. As shown in the color bar, red indicates up-regulation; green indicates down-regulation; black indicates no change; and grey indicates no data available. (C) The estrogen receptor (*ESR1*) and *ERBB2* oncogene were markedly up-regulated in luminal breast tumors (ER/PR+, HER2+/−). (D) *KRT5* and *KRT17* were markedly up-regulated in triple-negative breast tumors.

Prognostic impact of gene expression profiling has also been widely studied in human breast cancer, and various multi-gene signatures have been proposed for breast cancer prognosis [18]–[22]. However, they were proven to be clinically accurate only for hormone receptor positive cases. The underlying molecular mechanisms driving distant metastatic invasion of triple-negative breast cancer are poorly understood. This study thus aimed to establish prognostic correlations between gene expression profiling and recurrence outcome of triple-negative breast cancer using oligonucleotide microarray technology. A 45-gene prognostic module was found to be statistically predictive of recurrence outcome of triple-negative breast cancer. Functional network analysis of the 45 genes revealed that deregulated TGF-β immune/inflammatory signaling may profoundly participate in metastatic invasion of triple-negative breast cancer.

## Methods

### Selection of Breast Cancer Specimens

Specimens of breast cancer tissues were collected and snap-frozen from breast cancer patients who had surgery between 1995 and 2008 at National Taiwan University Hospital (NTUH, Taipei, Taiwan). Clinicopathological information was obtained for all breast cancer patients along with informed consent. The American Joint Committee on Cancer (AJCC) TNM system (version 6) was used for breast cancer staging classification. Histological classification of breast cancer was determined by professional pathologists. Treatment procedure of all breast cancer patients followed National Comprehensive Cancer Network (NCCN) guideline. All breast tumor samples were neoadjuvant-free and were collected before systemic chemotherapy treatments. Selection of breast tumor samples aimed to avoid bias. A total of 157 breast tumor specimens, including 51 triple-negative (ER-, PR-, HER2-) and 106 luminal (ER/PR+, HER2+/−) breast tumors, were selected for microarray analysis. All triple-negative breast tumors were invasive ductal carcinomas (IDC). Use of human breast tumors in microarray experiments was approved by the Institutional Review Board at NTUH, and was performed in accordance with the policies of the hospital.

**Figure 2:**
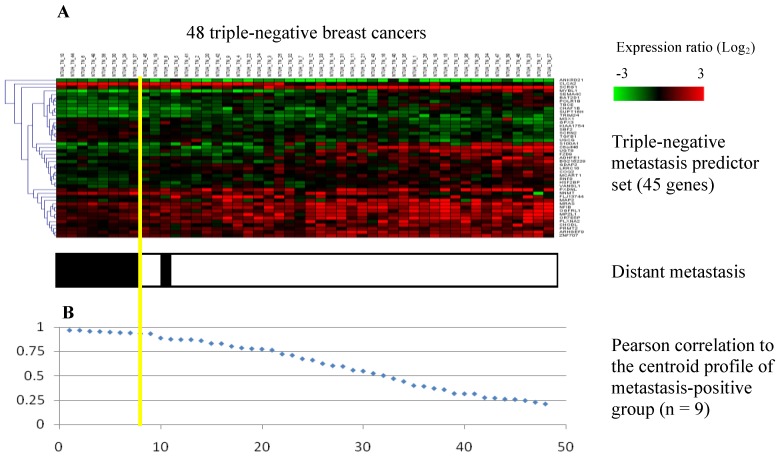
Classification of 48 triple-negative breast cancer patients using the 45-gene metastasis predictor set. (A) Gene expression data of the 45 genes from 48 triple-negative patients in a heat map. Each row represents a gene and each column represents a patient. Triple-negative breast cancer patients who developed distant metastases during the three years of follow-up were indicated with a black bar at the bottom of each column (white: distant-metastasis negative, black: distant-metastasis positive). The yellow line represents the metastasis predictor with optimal accuracy (sensitivity: 89%; specificity: 100%). The poor prognosis group (left) and the good prognosis group (right) were separated by the yellow line. (B) Rank-ordered Pearson correlation coefficients of 48 triple-negative patients with respect to the centroid profile of the metastasis-positive group (n = 9).

### Oligonucleotide Microarray Experiment

Total RNA from each specimen was extracted using Trizol Reagent (Invitrogen, Carlsbad, CA, USA) and then purified as the starting material for first strand cDNA synthesis (SuperScript II RNase H Reverse Transcriptase, Invitrogen) followed by second strand cDNA synthesis. The synthesized cDNA was used as templates to produce cRNA targets during the *in vitro* transcription process (MEGAscript T7 Kit, Applied Biosystems Ambion, Austin, TX, USA). A human reference RNA pool from 10 cell lines (Stratagene, La Jolla, CA, USA) served as the control reference in array signal comparison. RNA targets from tumor specimens were labeled with Cy5, and RNA reference targets were labeled with Cy3. The microarray platform used in this study was Agilent Human 1A (version 2) oligonucleotide microarray (Agilent Technologies, Santa Clara, CA, USA). After labeling and fragmentation, labeled cRNA targets from each tumor specimen and reference were pooled and hybridized to an oligonucleotide microarray at 60°C for 17 hours. Upon completion of hybridization, the arrays were washed and dried in nitrogen, and then scanned by an Agilent microarray scanner at 535 nm for Cy3 and at 625 nm for Cy5. Scanned images were analyzed using the feature extraction software (Agilent Technologies).

**Figure 3:**
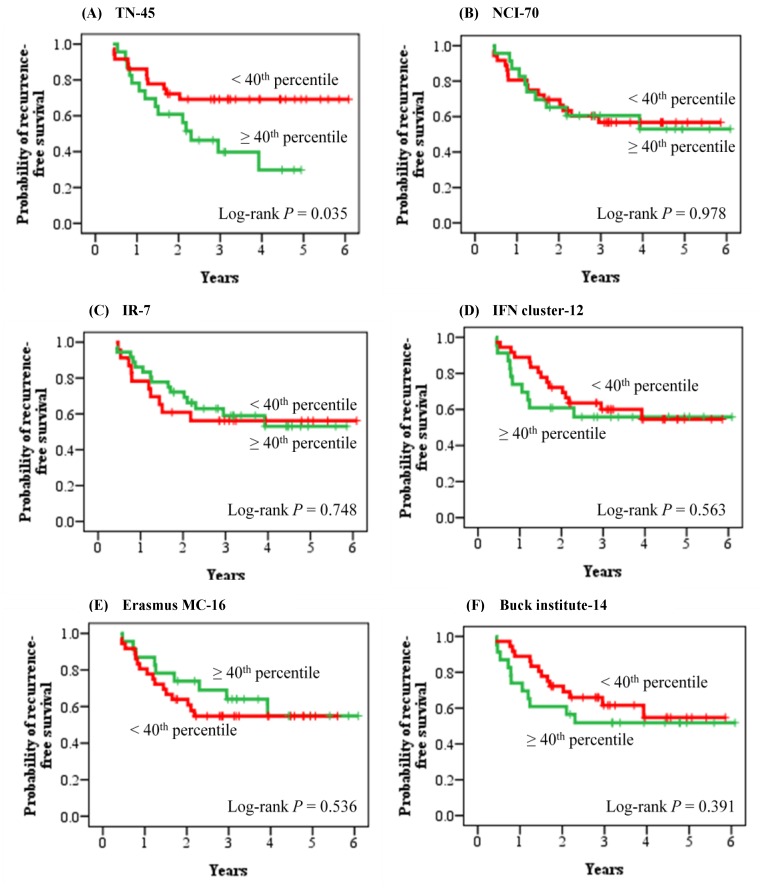
Comparative assessment of six breast cancer prognostic signatures. (A) TN-45, triple-negative metastasis predictor (45 genes). (B) NCI-70, breast cancer prognostic signature (70 genes) from the Netherlands Cancer Institute. (C) IR-7, immune response signature (7 genes). (D) IFN cluster-12, interferon cluster (12 genes). (E) Erasmus MC-16, breast cancer prognostic signature (16 genes) from Erasmus Medical Center. (F) Buck Institute-14, triple-negative metastasis predictor (14 genes) from Buck Institute. In the analysis of each multi-gene prognostic signature, 59 early-stage triple-negative patients from the validation cohort were first rank-ordered according to the proposed method in each study and then divided into two groups of opposite prognosis at the fortieth percentile cut-point. Patients with index values above the fortieth percentile cut-point were classified as the poor prognosis group (n = 23) and patients with index values below it were classified as the good prognosis group (n = 36).

### Microarray Data Analysis

Microarray data from 157 breast cancer samples were collected and organized in an Excel data sheet, with 20,140 genes eligible for analysis. All microarray data were deposited in the Gene Expression Omnibus [GEO:GSE33926] of the National Center for Biotechnology Information (NCBI, Bethesda, MD, USA). Gene expression data of 157 microarrays were normalized using quantile normalization. In the analysis of association between triple-negative and basal-like breast cancer, 261 intrinsic genes [12], [13] were first identified on our microarray platform. Average-linkage hierarchical clustering was performed on all 157 breast tumors using the intrinsic gene subset with Genesis software (version 1.7.4) (Figure S1).

**Figure 4:**
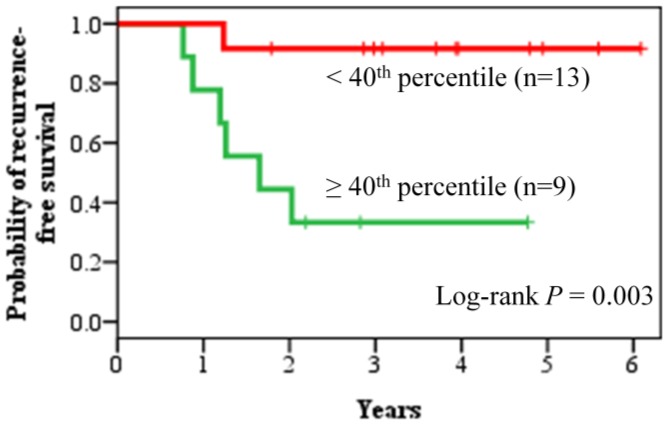
Prognostic performance of the metastasis predictor genes was evaluated with node-negative triple-negative patients. Recurrence-free survival analysis of 22 node-negative triple-negative patients in the validation cohort was performed using the metastasis predictor genes. The patients were divided into the good prognosis group (n = 13) and the poor prognosis group (n = 9) at the fortieth percentile cut-point.

In the search for metastasis predictor genes, 9 out of 48 triple-negative patients who developed distant metastases in the follow-up period were classified as the metastasis-positive group (n = 9; mean time to metastasis: 2.1±1.2 years), and the remaining 39 patients who were free of distant metastasis during the follow-up period were classified as the metastasis-negative group (n = 39). The median follow-up period of the triple-negative patients was 4.4 years. Clinicopathological information of the 48 triple-negative patients is shown in Table S1. Differentially expressed genes between the two groups were first identified by using the two-sided Student’s t-test (*P*<0.005) and fold change comparison (≥1.5 fold). Fifty significantly expressed genes were determined after performing the Benjamini and Hochberg method for multiple-testing correction. To establish a multi-gene signature, combinations of the fifty genes were tested by adding one gene at a time based on the fold change magnitude of each gene. Receiver operating characteristic (ROC) analysis was performed with each combination using the IBM SPSS software (version 16.0). The combination of 45 marker genes was found to provide the highest area under curve (AUC) value in prediction of distant recurrence (Figure S2). Leave-one-out cross validation was performed to examine the prediction power of the 45-gene signature using the support vector machine algorithm.

**Figure 5:**
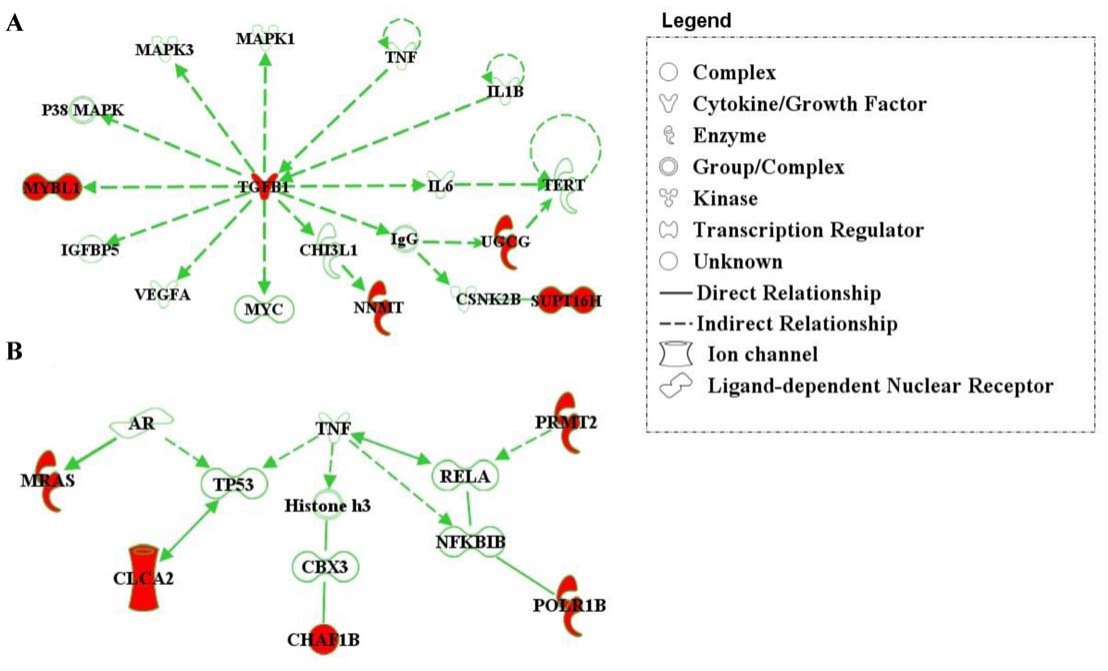
Molecular network analyses by IPA. Deregulated genes from the 45-gene signature are colored in red. The direction of the arrows indicates a functional relationship between an upstream regulator and a downstream element. (A) The cellular proliferation network comprising five metastasis predictor genes (*TGFB1*, *MYBL1*, *NNMT*, *UGCG*, and *SUPT16H*) from the 45-gene signature. (B) Network diagram linking five metastasis predictor genes (*MRAS*, *CLCA2*, *CHAF1B*, *POLR1B*, and *PRMT2*) associated with TNF regulatory pathway.

### External Validation

The prognostic performance of the 45-gene signature was validated using an independent microarray dataset [GEO:GSE25065] from the Gene Expression Omnibus. The validation cohort contained 59 early-stage triple-negative breast cancer patients annotated for recurrence events and relapse-free survival time. Microarray data of 59 triple-negative tumors were normalized using quantile normalization. Thirty-two out of the 45 predictor genes were identified in the validation microarray platform (Table S2). Kaplan-Meier survival curves of recurrence-free survival in good/poor prognosis groups of triple-negative patients were plotted using the IBM SPSS software.

### Pathway Analysis

Functional classification linked to the 45 metastasis predictor genes was identified by the Ingenuity Pathway Analysis (IPA) software (version 8.8). Gene symbols were used as input for the search of biological functions and molecular networks associated with the 45-gene signature. The contexts of functional analysis were delineated by gene ontology. Molecular networks harboring at least five metastasis predictor genes were selected and analyzed with the Ingenuity software.

## Results

### Clinicopathological Characteristics

Clinicopathological information of 157 invasive breast carcinomas composing the study sample is shown in Table 1. Forty-eight (94%) triple-negative patients were diagnosed at early stages (I-III) with no signs of distant metastasis. Association between the clinicopathological characteristics and triple-negative phenotype of breast cancer was investigated by compared with luminal breast cancer (Table S4). High histologic grade (*P*<0.001) was more commonly observed in triple-negative breast tumors (68.1%) than in luminal breast tumors (23.5%). High mitotic count (*P*<0.001) and high nuclear pleomorphism (*P*<0.001) were more prevalent in triple-negative breast cancer, as 44.7% and 78.7% of the triple-negative patients were diagnosed with high mitotic count and high nuclear pleomorphism, respectively, and 14.7% and 34.7% of the luminal breast cancer patients were with high mitotic count and high nuclear pleomorphism, respectively. Low tubule formation (*P* = 0.012; i.e., less than 10% carcinoma composed of tubular structure) was more commonly observed in triple-negative tumors (83.0%) than in luminal breast tumors (62.1%).

**Table 1:** Clinicopathological characteristics of 157 invasive breast carcinomas.

Characteristic	N	Triple-negative (n = 51)	Luminal (n = 106)
		n (%)	n (%)
**Age at diagnosis**	157		
<50		14 (27.5)	53 (50.0)
≥50		37 (72.5)	53 (50.0)
**Stage**	157		
I		12 (23.5)	17 (16.0)
II		25 (49.0)	45 (42.5)
III		11 (21.6)	38 (35.8)
IV		3 (5.9)	6 (5.7)
**Tumor size**	157		
1 (<2cm)		15 (29.4)	31 (29.2)
2 (2cm–5cm)		28 (54.9)	57 (53.8)
3 (>5cm)		8 (15.7)	15 (14.2)
4 (direct extension to chest wall or skin)		0	3 (2.8)
**Grade**	145		
1 (low)		0	24 (24.5)
2 (intermediate)		15 (31.9)	51 (52.0)
3 (high)		32 (68.1)	23 (23.5)
**Lymph node metastasis**	156		
Negative		30 (58.8)	32 (30.5)
Positive		21 (41.2)	73 (69.5)
**Lymphovascular invasion**	135		
Negative		22 (47.8)	29 (32.6)
Positive		24 (52.2)	60 (67.4)
**Mitotic count**	142		
1 (≤7)		10 (21.3)	54 (56.8)
2 (8–14)		16 (34.0)	27 (28.4)
3 (>14)		21 (44.7)	14 (14.7)
**Nuclear pleomorphism**	142		
1 (low)		0	8 (8.4)
2 (intermediate)		10 (21.3)	54 (56.8)
3 (high)		37 (78.7)	33 (34.7)
**Tubule formation**	142		
1 (>75%)		0	3 (3.2)
2 (10%–75%)		8 (17.0)	33 (34.7)
3 (<10%)		39 (83.0)	59 (62.1)

### Transcriptomic Portrait

Molecular similarities between triple-negative breast cancer and basal-like breast cancer were investigated at the mRNA expression level using the intrinsic genes previously determined as the molecular classifiers of luminal A, luminal B, HER2/neu, basal-like, and normal breast-like subtypes of breast cancer [12], [13]. Hierarchical clustering analysis was performed on 157 breast tumors with 261 intrinsic genes using the average-linkage clustering algorithm (Figure 1A and 1B). The breast tumors were distinctively classified into two dominant clusters based on the expression patterns of the intrinsic genes. The luminal cluster predominantly harboring luminal breast tumors (ER/PR+, HER2+/−) was associated with strong overexpression of the estrogen receptor (*ESR1*) and the *ERBB2* oncogene (Figure 1C). Forty-eight (94%) triple-negative breast tumors and 18 (17%) luminal breast tumors were clustered together, showing prominent basal-like characteristics where basal marker genes *KRT5* and *KRT17* were markedly up-regulated (Figure 1D).

### Metastasis Predictor Genes of Triple-negative Breast Cancer

Microarray data from the 48 triple-negative breast cancer patients (mean age: 54±11) who had no signs of distant metastasis at initial diagnosis were used for the search for metastasis predictor genes. The patients were divided into two groups (metastasis-positive vs. metastasis–negative) based on disease outcome. A panel of 45 significantly expressed genes (Table 2) was determined as the metastasis predictor set as described in the Methods section. The accuracy of prediction with the 45-gene signature was further evaluated by leave-one-out cross validation using the support vector machine algorithm, and 45 out of 48 patients were predicted correctly in distant recurrence outcome (accuracy: 94%). Centroid classification of 48 patients was performed using gene expression data of the 45-gene signature (Figure 2A). The 48 triple-negative patients were arranged in order according to their Pearson correlation coefficients to the centroid (mean expression) profile of the metastasis-positive group (Figure 2B). The yellow line represents the optimal classifier that predicted correctly the actual disease outcome for 47 out of 48 triple-negative patients (sensitivity: 89%; specificity: 100%; accuracy: 98%). Disease outcomes of 39 metastasis-negative patients and 8 metastasis-positive patients were correctly predicted, with only one metastasis-positive patient assigned incorrectly to the opposite category.

**Table 2:** 45-gene metastasis predictor module for triple-negative breast cancer.

Agilent probe	Gene symbol	Fold change	Gene description
**Up-regulated genes in triple-negative patients with poor prognosis**			
A_23_P420348	*ANKRD21*	2.1	POTE ankyrin domain family, member D
A_23_P397248	*CLCA2*	4.8	Chloride channel accessory 2
A_23_P133475	*GPX3*	1.9	Glutathione peroxidase 3 (plasma)
A_23_P340333	*KIAA1754*	1.6	Inositol 1,4,5-triphosphate receptor interacting protein
A_23_P110430	*MSX1*	1.6	Msh homeobox 1
A_23_P127584	*NNMT*	2.0	Nicotinamide N-methyltransferase
A_23_P98731	*SBF2*	1.5	SET binding factor 2
A_23_P4323	*SCRN2*	1.6	Secernin 2
A_23_P412335	*TGFB1*	1.5	Transforming growth factor, beta 1
A_23_P313380	*UGCG*	1.6	UDP-glucose ceramide glucosyltransferase
**Down-regulated genes in triple-negative patients with poor prognosis**			
A_23_P157569	*ADHFE1*	1.6	Alcohol dehydrogenase, iron containing, 1
A_23_P251701	*ARHGEF9*	1.7	Cdc42 guanine nucleotide exchange factor (GEF) 9
A_23_P412409	*BAT2D1*	1.5	Proline-rich coiled-coil 2C
A_23_P135123	*BG216229*	1.7	FERM domain containing 3
A_23_P353614	*C8orf46*	3.2	Chromosome 8 open reading frame 46
A_23_P57306	*CHAF1B*	2.0	Chromatin assembly factor 1, subunit B (p60)
A_23_P68669	*CHODL*	1.5	Chondrolectin
A_23_P113482	*COQ2*	1.5	Coenzyme Q2 homolog, prenyltransferase
A_23_P122655	*FLJ13744*	1.9	Hypothetical protein FLJ13744
A_23_P59613	*FZD9*	1.8	Frizzled homolog 9 (Drosophila)
A_23_P11915	*GDAP2*	1.5	Ganglioside induced differentiation associated protein 2
A_23_P143512	*HSF2BP*	1.5	Heat shock transcription factor 2 binding protein
A_23_P19364	*LRRC16*	1.6	Leucine rich repeat containing 16A
A_23_P153920	*MAP2*	2.2	Microtubule-associated protein 2
A_23_P112512	*MCART1*	1.5	Mitochondrial carrier triple repeat 1
A_23_P11874	*MPZL1*	2.0	Myelin protein zero-like 1
A_23_P41188	*MRAS*	1.5	Muscle RAS oncogene homolog
A_23_P43157	*MYBL1*	2.4	V-myb myeloblastosis viral oncogene homolog (avian)-like 1
A_23_P216448	*NFIB*	1.9	Nuclear factor I/B
A_23_P7791	*OGFRL1*	2.0	Opioid growth factor receptor-like 1
A_23_P20420	*OR7E5P*	1.8	Olfactory receptor, family 7, subfamily E, member 5 pseudogene
A_23_P307540	*PLXNA2*	1.7	Plexin A2
A_23_P209987	*POLR1B*	1.7	Polymerase (RNA) I polypeptide B, 128kDa
A_23_P211247	*PRMT2*	1.5	Protein arginine methyltransferase 2
A_23_P258310	*PXDNL*	2.2	Peroxidasin homolog (Drosophila)-like
A_23_P70384	*RNF8*	1.6	Ring finger protein 8
A_23_P383227	*S100A1*	3.0	S100 calcium binding protein A1
A_23_P167159	*SCRG1*	5.0	Stimulator of chondrogenesis 1
A_23_P114057	*SEMA4C*	1.6	Sema domain, immunoglobulin domain (Ig), transmembrane domain (TM) and shortcytoplasmic domain, (semaphorin) 4C
A_23_P151625	*SUPT16H*	1.5	Suppressor of Ty 16 homolog (S. cerevisiae)
A_23_P52147	*TBCE*	1.9	Tubulin folding cofactor E
A_23_P93629	*TRIM24*	1.5	Tripartite motif containing 24
A_23_P72747	*UGT8*	2.1	UDP glycosyltransferase 8
A_23_P103795	*VANGL1*	1.6	Vang-like 1 (van gogh, Drosophila)
A_23_P83714	*ZNF707*	1.5	Zinc finger protein 707

Fold change value was obtained by comparing the mean expression of each gene between the good and the poor prognosis groups of triple-negative patients (n = 48).

### External Validation

Prognostic performance of the 45-gene signature was further evaluated in an independent microarray dataset [GEO:GSE25065] that contains microarray data of 59 early-stage triple-negative breast cancer patients (AJCC stage I-IIIB; mean age: 49.5±10.9). This validation dataset was chosen because the immunohistochemical statuses of ER, PR, and HER2, clinical stage and recurrence information of all patients were completely documented. Twenty-five patients in this dataset who had disease recurrence (mean time to recurrence: 1.4±0.8 years) in the follow-up years were classified as the recurrence-positive group. Using gene expression data of our prognostic signature, 59 patients were rank-ordered based on correlation coefficients with the centroid profile of the recurrence-positive group. In order to ensure fair comparison, a cut-point at the fortieth percentile was chosen in correspondence with the actual recurrence rate. The 23 patients within the top 40% were classified as the poor prognosis group, and the remaining 36 patients were classified as the good prognosis group. Fourteen recurrence-positive cases were assigned correctly to the poor prognosis group, and 25 recurrence-negative cases were assigned correctly to the good prognosis group.

The predictive power of our 45-gene signature was compared with five other published breast cancer prognostic signatures in the same validation cohort using Kaplan-Meier survival analysis (Figure 3) and Cox hazard regression analysis (Table 3). The signatures used for comparative assessment were tested according to the published original methods. Kaplan-Meier survival analyses showed that, among the six breast cancer prognostic signatures, only our 45-gene signature was able to accurately predict the recurrence outcome of the 59 early-stage triple-negative patients (log-rank *P* = 0.035). The Cox proportional hazard ratio between the poor prognosis group and the good prognosis group, as predicted by our 45-gene signature, was 2.29 (95% CI 1.04–5.06). In addition, 22 node-negative triple-negative patients in the validation cohort were used again to evaluate the prognostic performance of our metastasis predictor gene set (Figure 4 and Table S3). The result showed that 18 (6 recurrence-positive patients and 12 recurrence-negative patients) out of 22 patients were assigned correctly to the prognostic categories, while 4 patients were predicted incorrectly (log-rank *P* = 0.003).

**Table 3:** Performance comparison of six breast cancer prognostic signatures in the validation cohort of 59 early-stage triple-negative breast cancer patients.

Breast cancer prognostic signature	Univariate Cox regression		Kaplan-Meier analysis
	Hazard ratio (95% CI)	Cox *P* value	Log-rank *P* value
TN-45	2.29 (1.04–5.06)	0.040	0.035
NCI-70 [18]	1.01 (0.45–2.25)	0.978	0.978
IR-7 [19]	1.14 (0.51–2.54)	0.748	0.748
IFN cluster-12 [20]	1.27 (0.57–2.82)	0.564	0.563
Erasmus MC-16 [21]	0.77 (0.34–1.75)	0.537	0.536
Buck Institute-14 [22]	1.41 (0.64–3.11)	0.393	0.391

CI, confidence interval; TN-45, triple-negative metastasis predictor (45 genes); NCI-70, breast cancer prognostic signature (70 genes) from the Netherlands Cancer Institute; IR-7, immune response signature (7 genes); IFN cluster-12: interferon cluster (12 genes); Erasmus MC-16, breast cancer prognostic signature from Erasmus Medical Center (16 genes); Buck Institute-14, triple-negative metastasis predictor from Buck Institute (14 genes).

### Functional Network Analysis

The underlying biological mechanisms of the 45-gene metastasis predictor set were investigated by IPA. A molecular network incorporating five marker genes (*TGFB1*, *MYBL1*, *SUPT16H*, *NNMT*, and *UGCG*) that promotes proliferation of triple-negative breast tumors was identified by the Ingenuity software (Figure 5A). *TGFB1* is a common regulator of a variety of downstream kinases, growth factors, and transcription factors, including mitogen-activated protein kinase 1, mitogen-activated protein kinase 3, P38MAPK complex, MYC, insulin-like growth factor binding protein 5, vascular endothelial growth factor A, IgG, CHI3LI, and interleukin 6. Deregulation of MYC, vascular endothelial growth factor A, and interleukin 6 has been reported to be strongly associated with tumor growth [23], [24] and metastatic invasion [25], [26]. Five marker genes (*CHAF1B*, *CLCA2*, *MRAS*, *POLR1B*, and *PRMT2*) were found to be involved in the tumor necrosis factor (TNF) regulatory network (Figure 5B).

## Discussion

Clinicopathological information gathered from 51 triple-negative and 106 luminal breast cancers in this study showed that triple-negative breast cancer was markedly associated with high histological grade (*P*<0.001), high mitotic count (*P*<0.001), strong nuclear pleomorphism (*P*<0.001), and low tubule formation (*P* = 0.012). These clinicopathological characteristics identified in our triple-negative samples have been similarly documented in previous studies of basal-like breast cancer [2], [8], [11]. Livasy *et al.* reported that basal-like breast cancer was significantly associated with high histologic grade, lymphocytic infiltrate, and high mitotic counts in a morphologic evaluation of 23 basal-like carcinomas [8]. In addition, triple-negative tumors had larger variations in nuclear shape and size as compared with luminal breast tumors, as 78.7% of triple-negative tumors were diagnosed as having high nuclear pleomorphism, while only 34.7% of luminal breast tumors had high nuclear pleomorphism in our data.

Hierarchical clustering analysis of 157 breast cancers using the intrinsic gene subset also revealed that 94% of triple-negative tumors were clustered together, harboring high expression of basal marker genes and low expression of the *ESR1* gene. Several basal-related genes directly corresponding to expression levels of cytokeratins and EGFR protein marker, including *KRT5*, *KRT6C*, *KRT6E*, *KRT14*, and *EGFR* genes, were found to be extensively up-regulated in triple-negative breast cancer (Figure S3). However, triple-negative breast cancer is not a complete surrogate of basal-like breast cancer, as 6% of triple-negative tumors were sparsely intertwined within the luminal-rich cluster carrying low basal-like mRNA expression in our data.

Association between each clinical variable and metastasis outcome of triple-negative breast cancer was analyzed in our dataset by using the Fisher’s exact test (Table S1) and Cox regression model for univariate and multivariate analyses (Table S5). None of the traditional measures were found to be statistically prognostic for distant recurrence of triple-negative breast cancer in our data. The disease outcome of 48 triple-negative patients in our training cohort showed that lymph node status was not a strong clinical determinant of distant metastasis recurrence (Table S1; distant metastasis-positive group (6 node-positive and 3 node-negative) versus distant metastasis-negative group (13 node-positive and 26 node-negative); Fisher’s exact test *P* = 0.073). Both node-positive and node-negative triple-negative patients were included in our sample body in the assessment of prognostic genes for distant metastatic invasion of triple-negative breast cancer.

The functional pathway analysis revealed that the 45-gene signature was mostly characterized by genes related to cellular proliferation, cell cycle, and DNA replication. The *MRAS* gene encodes the M-Ras protein, which directly interacts with androgen receptor in the cell cycle and cellular development pathways. The *CLCA2* gene interacts with *ITGB4*, which also participates in cellular growth and the cell cycle. The *CHAF1B* and *SBF2* genes are involved in DNA replication, recombination, and repair pathways. The *TGFB1*, *MYBL1*, *NNMT*, *SUPT16H*, and *UGCG* genes are within a focused cellular proliferation network regulated by cytokines *TNF* and *IL1B*. Several downstream effectors of *TGFB1* are known for oncogenic characteristics, including vascular endothelial growth factor A, c-Myc (MYC), c-Jun N-terminal kinase (JNK), mitogen-activated protein kinases, and P38MAP complex [27]. Thus it appears that many genes in our 45-gene prognostic module, and their downstream targets, are predominantly involved in biological functions promoting tumor progression.

This study documented the first evidence that deregulated genes within the TGF-β signaling pathway were markedly involved in distant recurrence of triple-negative breast cancer. The TGF-β signaling pathway has been reported as a key regulator of the epithelial-to-mesenchymal transition (EMT) process that converts primary tumors to metastases in advanced malignancy of human cancers [28]–[31]. Overexpression of *TGFB1* in those triple-negative tumors with poor prognosis may thus induce EMT and enhance the aggressiveness of tumor behavior. Noticeably, *TNF* and *IL1B*, two upstream regulators of *TGFB1*, are mediators of immune/inflammatory response, and *TGFB1* itself is particularly crucial in the regulation of T cell-mediated immune mechanisms [32]. A total of ten differentially expressed genes in our 45-gene module were found to be within the TNF regulatory pathway. Altogether, our data suggests that distant metastatic invasion of triple-negative breast cancer may be potentially induced by immune/inflammatory deregulation.

To our knowledge, most multi-gene prognostic signatures that have been proposed to date for prediction of breast cancer recurrence were not tailored for triple-negative patients. To be noticed that in our comparative assessment, NCI-70 was derived from primary breast tumors and IR-7 and Erasmus MC-16 were derived from ER- patient cohorts. The Buck institute-14 signature was derived from 199 hormone-negative breast cancer cases curated from three microarray datasets, and 154 ER-HER2- patients of them were claimed to be triple-negative cases despite the lack of PR status. The 45-gene prognostic signature identified in this study was derived from a homogeneous population of triple-negative patients, thus giving less opportunity for confounding by breast cancer heterogeneity.

According to the St Gallen consensus for chemotherapy guidelines, all triple-negative patients are recommended to receive adjuvant systemic chemotherapy. For example, a combination of anthracycline-based regimens with taxanes is a common treatment option for triple-negative patients. However, this combination of treatment regimens often brings serious side effects to the patients. Our 45-gene prognostic predictor, if further confirmed, may become a clinically useful tool that identifies triple-negative patients at low risk of distant recurrence, allowing them to receive moderate doses of the combined regimens or even only the anthracycline-based regimens to reduce side effects for the patients. Recently a number of pathway target agents, including EGFR inhibitors, DNA repair pathway inhibitors, anti-angiogenic agents, etc., are under vigorous clinical trials for target therapies of triple-negative breast cancer [33], and they may be used along with traditional chemotherapy treatment for those triple-negative patients with unfavorable prognosis. On the other hand, currently the presence of axillary lymph node metastasis of a triple-negative patient is considered to be determinant for giving aggressive chemotherapy (third-generation chemotherapy; i.e., paclitaxel, docetaxel, gemcitabine, etc.). However, it is unable to identify those node-negative triple-negative patients who are at high risk of developing distant metastasis, and those patients are in need of aggressive chemotherapy treatment. Our 45-gene prognostic signature may help identify this group of triple-negative patients and assist in treatment recommendation to enhance their chances of survival.

## Supporting Information

Figure S1 Hierarchical clustering diagram of 157 breast cancers (51 triple-negative and 106 luminal breast cancers) using the 261 intrinsic genes. Each row represents a gene and each column represents a breast tumor. On top of each column, tumors were marked with red and blue to indicate triple-negative and luminal subtypes of breast cancers, respectively. Genes and tumor samples were clustered together according to their similarities of expression patterns as depicted by the dendrogram.
(PDF)

Figure S2 Establishment of the 45-gene prognostic predictor set for triple-negative breast cancer. (A) Area under the curve (AUC) values of each receiver operating characteristic (ROC) curve obtained with different numbers of genes ranked by the magnitude of fold change between the metastasis-positive group (n = 9) and the metastasis-negative group (n = 39). (B) ROC curve of the 45-gene prognostic signature with optimal AUC value (0.994).
(PDF)

Figure S3 Distributions of gene expression intensities of five basal marker genes (KRT5, KRT6C, KRT6E, KRT14, and EGFR) within triple-negative (n = 51) and luminal (n = 106) breast cancers. The P value of each basal marker gene was calculated with the two-sided Student’s t-test. Mean±SD were shown.
(PDF)

Table S1 Association between clinical characteristics and metastasis outcome of 48 triple-negative breast cancer patients in our dataset were investigated. The P values were calculated by using the Fisher’s exact test.
(PDF)

Table S2 Affymetrix probe ID, gene symbol, and description of 32 metastasis predictor genes identified in the validation dataset [GEO:GSE25065].
(PDF)

Table S3 Clinical characteristics, recurrence information, and Pearson correlation coefficient (with respect to the recurrence-positive group (n = 7) using the 32 metastasis predictor genes) of 22 node-negative triple-negative breast cancer patients in the validation dataset [GEO:GSE25065].
(PDF)

Table S4 Association between clinical features and triple-negative phenotype of breast cancer as compared with luminal breast cancer.
(PDF)

Table S5 Univariate and multivariate analyses for distant-metastasis-free survival were performed with each prognostic factor in our triple-negative patient dataset by using the Cox regression model. The multivariate analysis included 45 triple-negative breast cancer patients, owing to missing values in 3 patients.
(PDF)

## References

[1] Cleator S, Heller W, Coombes RC (2007) Triple-negative breast cancer: therapeutic options. Lancet Oncol 8: 235–244. 17329194 10.1016/S1470-2045(07)70074-8

[2] Foulkes WD, Smith IE, Reis-Filho JS (2010) Triple-negative breast cancer. N Engl J Med 363: 1938–1948. 21067385 10.1056/NEJMra1001389

[3] Rakha EA, El-Sayed ME, Green AR, Lee AH, Robertson JF, et al (2007) Prognostic markers in triple-negative breast cancer. Cancer 109: 25–32. 17146782 10.1002/cncr.22381

[4] Stead LA, Lash TL, Sobieraj JE, Chi DD, Westrup JL, et al (2009) Triple-negative breast cancers are increased in black women regardless of age or body mass index. Breast Cancer Res 11: R18. 19320967 10.1186/bcr2242 PMC2688946

[5] Lund MJ, Trivers KF, Porter PL, Coates RJ, Leyland-Jones B, et al (2009) Race and triple negative threats to breast cancer survival: a population-based study in Atlanta, GA. Breast Cancer Res Treat 113: 357–370. 18324472 10.1007/s10549-008-9926-3

[6] Bauer KR, Brown M, Cress RD, Parise CA, Caggiano V (2007) Descriptive analysis of estrogen receptor (ER)-negative, progesterone receptor (PR)-negative, and HER2-negative invasive breast cancer, the so-called triple-negative phenotype: a population-based study from the California cancer Registry. Cancer 109: 1721–1728. 17387718 10.1002/cncr.22618

[7] Dent R, Trudeau M, Pritchard KI, Hanna WM, Kahn HK, et al (2007) Triple-negative breast cancer: clinical features and patterns of recurrence. Clin Cancer Res 13: 4429–4434. 17671126 10.1158/1078-0432.CCR-06-3045

[8] Livasy CA, Karaca G, Nanda R, Tretiakova MS, Olopade OI, et al (2006) Phenotypic evaluation of the basal-like subtype of invasive breast carcinoma. Mod Pathol 19: 264–271. 16341146 10.1038/modpathol.3800528

[9] Nielsen TO, Hsu FD, Jensen K, Cheang M, Karaca G, et al (2004) Immunohistochemical and clinical characterization of the basal-like subtype of invasive breast carcinoma. Clin Cancer Res 10: 5367–5374. 15328174 10.1158/1078-0432.CCR-04-0220

[10] Kreike B, van Kouwenhove M, Horlings H, Weigelt B, Peterse H, et al (2007) Gene expression profiling and histopathological characterization of triple-negative/basal-like breast carcinomas. Breast Cancer Res 9: R65. 17910759 10.1186/bcr1771 PMC2242660

[11] Tan DS, Marchio C, Jones RL, Savage K, Smith IE, et al (2008) Triple negative breast cancer: molecular profiling and prognostic impact in adjuvant anthracycline-treated patients. Breast Cancer Res Treat 111: 27–44. 17922188 10.1007/s10549-007-9756-8

[12] Perou CM, Sorlie T, Eisen MB, van de Rijn M, Jeffrey SS, et al (2000) Molecular portraits of human breast tumours. Nature 406: 747–752. 10963602 10.1038/35021093

[13] Sorlie T, Tibshirani R, Parker J, Hastie T, Marron JS, et al (2003) Repeated observation of breast tumor subtypes in independent gene expression data sets. Proc Natl Acad Sci U S A 100: 8418–8423. 12829800 10.1073/pnas.0932692100 PMC166244

[14] Cheang MC, Voduc D, Bajdik C, Leung S, McKinney S, et al (2008) Basal-like breast cancer defined by five biomarkers has superior prognostic value than triple-negative phenotype. Clin Cancer Res 14: 1368–1376. 18316557 10.1158/1078-0432.CCR-07-1658

[15] Rakha EA, Elsheikh SE, Aleskandarany MA, Habashi HO, Green AR, et al (2009) Triple-negative breast cancer: distinguishing between basal and nonbasal subtypes. Clin Cancer Res 15: 2302–2310. 19318481 10.1158/1078-0432.CCR-08-2132

[16] Rakha EA, Tan DS, Foulkes WD, Ellis IO, Tutt A, et al.. (2007) Are triple-negative tumours and basal-like breast cancer synonymous? Breast Cancer Res 9: 404; author reply 405. 10.1186/bcr1827 PMC2246182 18279542

[17] Yang XR, Sherman ME, Rimm DL, Lissowska J, Brinton LA, et al (2007) Differences in risk factors for breast cancer molecular subtypes in a population-based study. Cancer Epidemiol Biomarkers Prev 16: 439–443. 17372238 10.1158/1055-9965.EPI-06-0806

[18] van't Veer LJ, Dai H, van de Vijver MJ, He YD, Hart AA, et al (2002) Gene expression profiling predicts clinical outcome of breast cancer. Nature 415: 530–536. 11823860 10.1038/415530a

[19] Teschendorff AE, Miremadi A, Pinder SE, Ellis IO, Caldas C (2007) An immune response gene expression module identifies a good prognosis subtype in estrogen receptor negative breast cancer. Genome Biol 8: R157. 17683518 10.1186/gb-2007-8-8-r157 PMC2374988

[20] Hu Z, Fan C, Oh DS, Marron JS, He X, et al (2006) The molecular portraits of breast tumors are conserved across microarray platforms. BMC Genomics 7: 96. 16643655 10.1186/1471-2164-7-96 PMC1468408

[21] Wang Y, Klijn JG, Zhang Y, Sieuwerts AM, Look MP, et al (2005) Gene-expression profiles to predict distant metastasis of lymph-node-negative primary breast cancer. Lancet 365: 671–679. 15721472 10.1016/S0140-6736(05)17947-1

[22] Yau C, Esserman L, Moore DH, Waldman F, Sninsky J, et al (2010) A multigene predictor of metastatic outcome in early stage hormone receptor-negative and triple-negative breast cancer. Breast Cancer Res 12: R85. 20946665 10.1186/bcr2753 PMC3096978

[23] Frank NY, Schatton T, Kim S, Zhan Q, Wilson BJ, et al (2011) VEGFR-1 expressed by malignant melanoma-initiating cells is required for tumor growth. Cancer Res 71: 1474–1485. 21212411 10.1158/0008-5472.CAN-10-1660 PMC3083845

[24] Smola MG, Estelberger W, Reiter M, Schauenstein K, Schauenstein E (1991) Sigma S, a measure of reactive sulfur groups of immunoglobulin G, is a sensitive tumor marker discriminating different stages of breast cancer. Cancer 68: 1026–1030. 1913474 10.1002/1097-0142(19910901)68:5<1026::aid-cncr2820680520>3.0.co;2-h

[25] Wolfer A, Ramaswamy S (2011) MYC and metastasis. Cancer Res 71: 2034–2037. 21406394 10.1158/0008-5472.CAN-10-3776 PMC3089000

[26] Rapp UR, Korn C, Ceteci F, Karreman C, Luetkenhaus K, et al (2009) MYC is a metastasis gene for non-small-cell lung cancer. PLoS One 4: e6029. 19551151 10.1371/journal.pone.0006029 PMC2696940

[27] Derynck R, Zhang YE (2003) Smad-dependent and Smad-independent pathways in TGF-beta family signalling. Nature 425: 577–584. 14534577 10.1038/nature02006

[28] Zavadil J, Bottinger EP (2005) TGF-beta and epithelial-to-mesenchymal transitions. Oncogene 24: 5764–5774. 16123809 10.1038/sj.onc.1208927

[29] Eger A, Stockinger A, Park J, Langkopf E, Mikula M, et al (2004) beta-Catenin and TGFbeta signalling cooperate to maintain a mesenchymal phenotype after FosER-induced epithelial to mesenchymal transition. Oncogene 23: 2672–2680. 14755243 10.1038/sj.onc.1207416

[30] Bates RC, Mercurio AM (2003) Tumor necrosis factor-alpha stimulates the epithelial-to-mesenchymal transition of human colonic organoids. Mol Biol Cell 14: 1790–1800. 12802055 10.1091/mbc.E02-09-0583 PMC165077

[31] Fischer AN, Herrera B, Mikula M, Proell V, Fuchs E, et al (2005) Integration of Ras subeffector signaling in TGF-beta mediated late stage hepatocarcinogenesis. Carcinogenesis 26: 931–942. 15705598 10.1093/carcin/bgi043

[32] Liu Y, Teige I, Birnir B, Issazadeh-Navikas S (2006) Neuron-mediated generation of regulatory T cells from encephalitogenic T cells suppresses EAE. Nat Med 12: 518–525. 16633347 10.1038/nm1402

[33] Hudis CA, Gianni L (2011) Triple-negative breast cancer: an unmet medical need. Oncologist 16 Suppl 1:1–11. 10.1634/theoncologist.2011-S1-01 21278435

