# Supplementary figures and images for: Molecular Characteristics and Metastasis Predictor Genes of Triple-Negative Breast Cancer: A Clinical Study of Triple-Negative Breast Carcinomas
Source: PLoS One. 2012 Sep 25;7(9):e45831. doi: 10.1371/journal.pone.0045831 (PMC3458056; doi:10.1371/journal.pone.0045831)

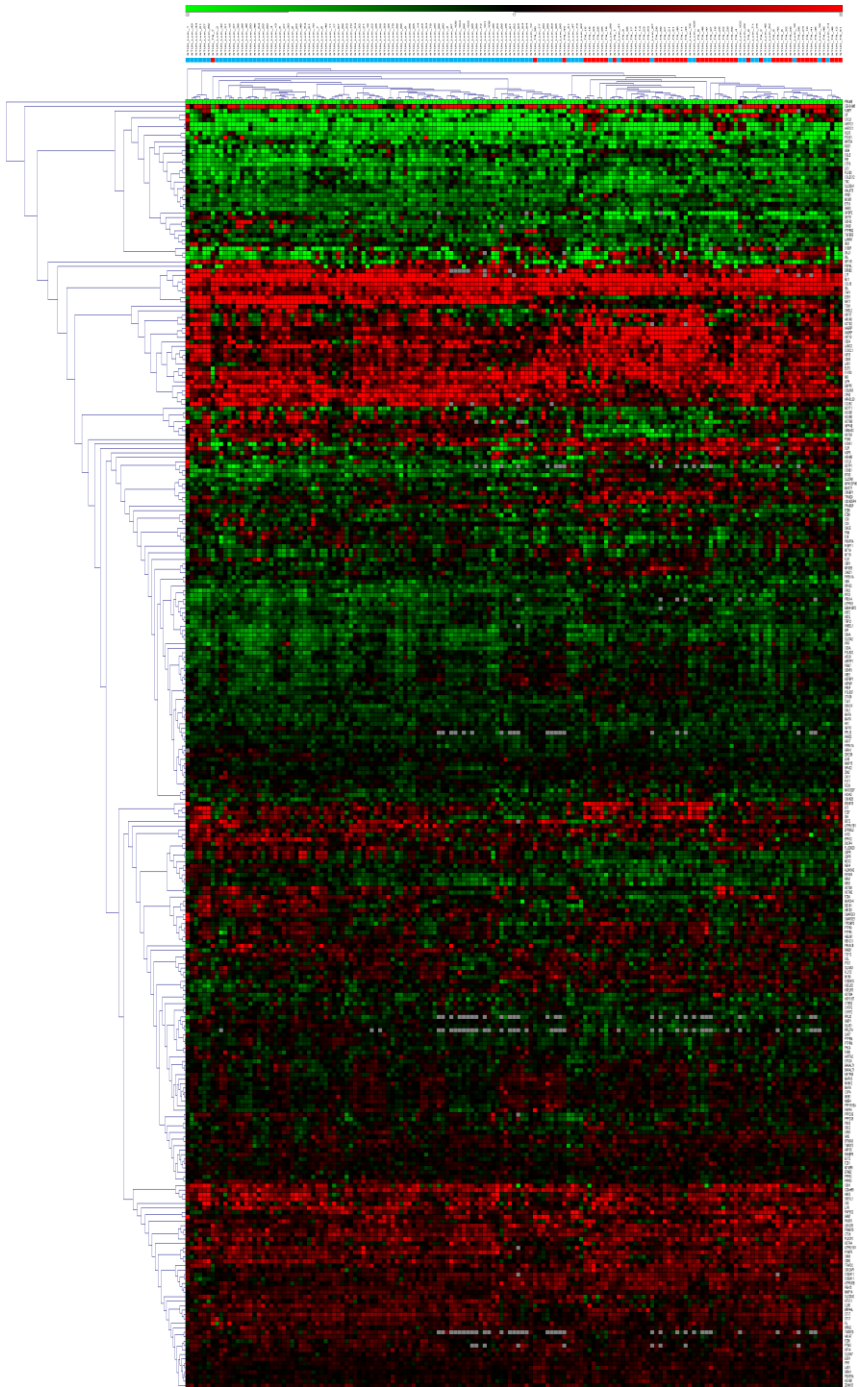

Supplement: Figure S1 — Hierarchical clustering diagram of 157 breast cancers (51 triple-negative and 106 luminal breast cancers) using the 261 intrinsic genes. Each row represents a gene and each column represents a breast tumor. On top of each column, tumors were marked with red and blue to indicate triple-negative and luminal subtypes of breast cancers, respectively. Genes and tumor samples were clustered together according to their similarities of expression patterns as depicted by the dendrogram. (PDF) [file pone.0045831.s001.pdf]

**A**

**Selection of prognostic marker genes**

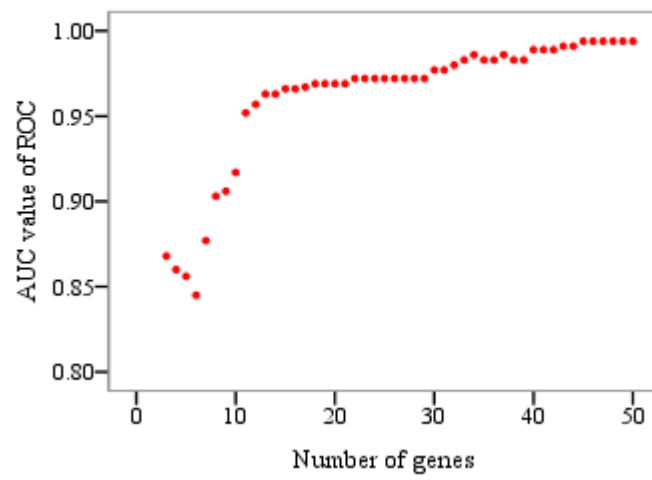

**B**

**ROC curve of 45-gene prognostic signature**

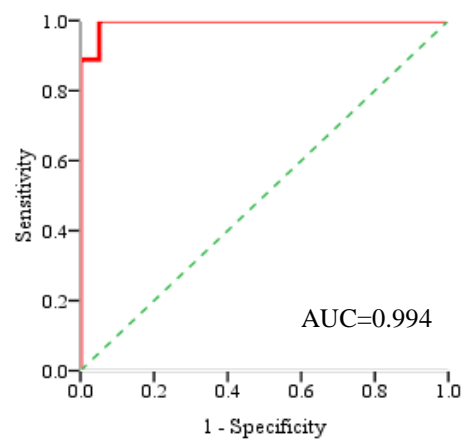

Supplement: Figure S2 — Establishment of the 45-gene prognostic predictor set for triple-negative breast cancer. (A) Area under the curve (AUC) values of each receiver operating characteristic (ROC) curve obtained with different numbers of genes ranked by the magnitude of fold change between the metastasis-positive group (n = 9) and the metastasis-negative group (n = 39). (B) ROC curve of the 45-gene prognostic signature with optimal AUC value (0.994). (PDF) [file pone.0045831.s002.pdf]

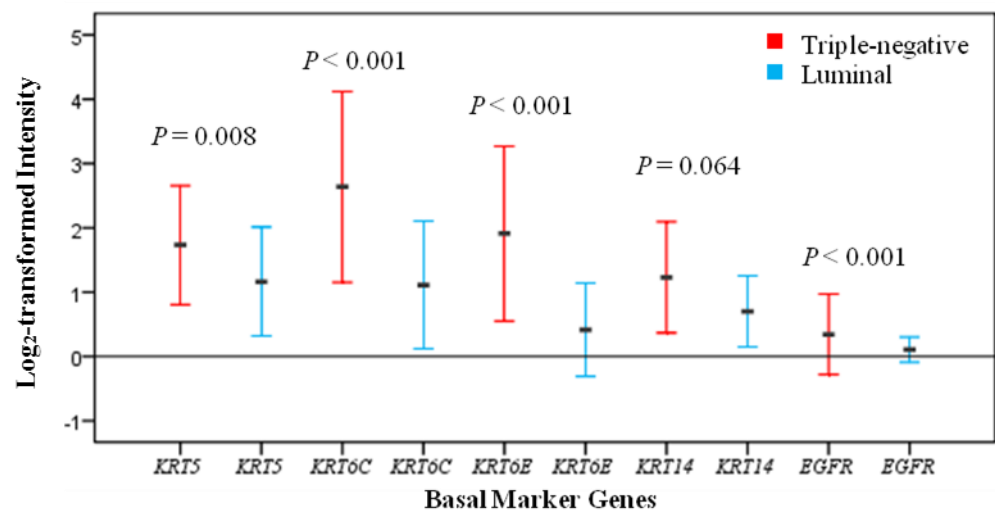

Supplement: Figure S3 — Distributions of gene expression intensities of five basal marker genes (KRT5, KRT6C, KRT6E, KRT14, and EGFR) within triple-negative (n = 51) and luminal (n = 106) breast cancers. The P value of each basal marker gene was calculated with the two-sided Student’s t-test. Mean±SD were shown. (PDF) [file pone.0045831.s003.pdf]
