# Supplementary material for: Molecular Characteristics and Metastasis Predictor Genes of Triple-Negative Breast Cancer: A Clinical Study of Triple-Negative Breast Carcinomas
Source: PLoS One. 2012 Sep 25;7(9):e45831. doi: 10.1371/journal.pone.0045831 (PMC3458056; doi:10.1371/journal.pone.0045831)
Supplement: Table S1 — Association between clinical characteristics and metastasis outcome of 48 triple-negative breast cancer patients in our dataset were investigated. The P values were calculated by using the Fisher’s exact test. (PDF) [file pone.0045831.s004.pdf]

Characteristic	N	Distant metastasis-positive n (%)	Distant metastasis-negative n (%)	*P* value
**Age at diagnosis**	48			0.090
< 50		0	13 (33.3)	
≥ 50		9 (100.0)	26 (66.7)	
**Stage**	48			0.106
I/II		5 (55.6)	32 (82.1)	
III		4 (44.4)	7 (17.9)	
**Tumor size**	48			0.578
≤5cm		7 (77.8)	35 (89.7)	
> 5cm		2 (22.2)	4 (10.3)	
**Grade**	45			0.542
Low/Intermediate		3 (37.5)	12 (32.4)	
High		5 (62.5)	25 (67.6)	
**Lymph node metastasis**	48			0.073
Negative		3 (33.3)	26 (66.7)	
Positive		6 (66.7)	13 (33.3)	
**Lymphovascular invasion**	44			0.206
Negative		2 (28.6)	20 (54.1)	
Positive		5 (71.4)	17 (45.9)	
**Mitotic count**	45			0.513
≤14		4 (50.0)	21 (56.7)	
> 14		4 (50.0)	16 (43.2)	
**Nuclear pleomorphism**	45			0.579
Low/Intermediate		2 (25.0)	8 (21.6)	
High		6 (75.0)	29 (78.4)	
**Tubule formation**	45			0.439
≥10%		2 (25.0)	6 (16.2)	
< 10%		6 (75.0)	31 (83.8)	
