# Supplementary material for: Molecular Characteristics and Metastasis Predictor Genes of Triple-Negative Breast Cancer: A Clinical Study of Triple-Negative Breast Carcinomas
Source: PLoS One. 2012 Sep 25;7(9):e45831. doi: 10.1371/journal.pone.0045831 (PMC3458056; doi:10.1371/journal.pone.0045831)
Supplement: Table S2 — Affymetrix probe ID, gene symbol, and description of 32 metastasis predictor genes identified in the validation dataset [GEO:GSE25065]. (PDF) [file pone.0045831.s005.pdf]

Affymetrix probe ID	Gene symbol	Gene description
203264_s_at	*ARHGEF9*	Cdc42 guanine nucleotide exchange factor (GEF) 9
214052_x_at	*BAT2D1*	CDNA FLJ20183 fis, clone COLF0283
204775_at	*CHAF1B*	Chromatin assembly factor 1, subunit B (p60)
219867_at	*CHODL*	Chondrolectin
206165_s_at	*CLCA2*	Chloride channel regulator 2
213379_at	*COQ2*	Coenzyme Q2 homolog, prenyltransferase
207639_at	*FZD9*	Frizzled homolog 9 (Drosophila)
219473_at	*GDAP2*	Ganglioside induced differentiation associated protein 2
201348_at	*GPX3*	Glutathione peroxidase 3 (plasma)
207020_at	*HSF2BP*	Heat shock transcription factor 2 binding protein
210015_s_at	*MAP2*	Microtubule-associated protein 2
201874_at	*MPZL1*	Myelin protein zero-like 1
206538_at	*MRAS*	Muscle RAS oncogene homolog
205932_s_at	*MSX1*	Msh homeobox 1
213906_at	*MYBL1*	V-myb myeloblastosis viral oncogene homolog (avian)-like 1
211466_at	*NFIB*	Nuclear factor I/B
202238_s_at	*NNMT*	Nicotinamide N-methyltransferase
219582_at	*OGFRL1*	Opioid growth factor receptor-like 1
213030_s_at	*PLXNA2*	Plexin A2
220113_x_at	*POLR1B*	Polymerase (RNA) I polypeptide B, 128kDa
202098_s_at	*PRMT2*	Protein arginine methyltransferase 2
203160_s_at	*RNF8*	Ring finger protein 8
205334_at	*S100A1*	S100 calcium binding protein A1
205475_at	*SCRG1*	Scrapie responsive protein 1
219039_at	*SEMA4C*	Sema domain, immunoglobulin domain (Ig), transmembrane domain (TM) and short cytoplasmic domain, (semaphorin) 4C
217815_at	*SUPT16H*	Suppressor of Ty 16 homolog (S. cerevisiae)
203715_at	*TBCE*	Tubulin folding cofactor E
203084_at	*TGFB1*	Transforming growth factor, beta 1
213301_x_at	*TRIM24*	Tripartite motif-containing 24
221765_at	*UGCG*	UDP-glucose ceramide glucosyltransferase
208358_s_at	*UGT8*	UDP glycosyltransferase 8
219330_at	*VANGL1*	Vang-like 1 (van gogh, Drosophila)
