# Supplementary material for: Molecular Characteristics and Metastasis Predictor Genes of Triple-Negative Breast Cancer: A Clinical Study of Triple-Negative Breast Carcinomas
Source: PLoS One. 2012 Sep 25;7(9):e45831. doi: 10.1371/journal.pone.0045831 (PMC3458056; doi:10.1371/journal.pone.0045831)
Supplement: Table S3 — Clinical characteristics, recurrence information, and Pearson correlation coefficient (with respect to the recurrence-positive group (n = 7) using the 32 metastasis predictor genes) of 22 node-negative triple-negative breast cancer patients in the validation dataset [GEO:GSE25065]. (PDF) [file pone.0045831.s006.pdf]

Specimen ID	Age (years)	Clinical T stage	Clinical nodal status	Clinical AJCC stage	Grade	Recurrence status	Recurrence-free (years)	Pearson correlation coefficient
GSM615640	49.0	T3	N0	IIB	3	1	1.19	0.89
GSM615733	64.0	T2	N0	IIA	3	0	2.19	0.85
GSM615739	53.0	T3	N0	IIB	3	1	1.65	0.82
GSM615650	60.1	T3	N0	IIB	3	0	4.77	0.82
GSM615687	39.0	T3	N0	IIB	3	1	1.26	0.81
GSM615800	40.0	T4	N0	IIIB	3	1	0.88	0.80
GSM615689	40.0	T3	N0	IIB	3	1	2.03	0.80
GSM615757	62.6	T1	N0	I	2	1	0.76	0.78
GSM615715	50.0	T2	N0	IIA	3	0	2.82	0.78
GSM615793	53.0	T4	N0	IIIB	NA	0	2.98	0.77
GSM615681	52.8	T2	N0	IIA	NA	1	1.23	0.76
GSM615707	63.0	T2	N0	IIA	3	0	4.79	0.76
GSM615672	49.6	T3	N0	IIB	NA	0	2.86	0.74
GSM615637	34.1	T3	N0	IIB	3	0	4.94	0.73
GSM615763	45.2	T2	N0	IIA	3	0	6.09	0.72
GSM615828	45.0	T2	N0	IIA	1	0	3.08	0.71
GSM615744	48.0	T2	N0	IIA	3	0	1.79	0.71
GSM615813	68.0	T3	N0	IIB	3	0	3.70	0.63
GSM615764	61.8	T2	N0	IIA	3	0	5.59	0.63
GSM615824	46.0	T2	N0	IIA	3	0	3.18	0.61
GSM615680	48.4	T3	N0	IIB	3	0	3.93	0.59
GSM615671	38.6	T3	N0	IIB	2	0	3.96	0.56
