# Supplementary material for: Molecular Characteristics and Metastasis Predictor Genes of Triple-Negative Breast Cancer: A Clinical Study of Triple-Negative Breast Carcinomas
Source: PLoS One. 2012 Sep 25;7(9):e45831. doi: 10.1371/journal.pone.0045831 (PMC3458056; doi:10.1371/journal.pone.0045831)
Supplement: Table S4 — Association between clinical features and triple-negative phenotype of breast cancer as compared with luminal breast cancer. (PDF) [file pone.0045831.s007.pdf]

Characteristic	N	Triple-negative breast cancer	Luminal breast cancer	*P* value
		n (%)	n (%)	
**Age at diagnosis**	157			0.010*
< 50		14 (27.5)	53 (50.0)	
≥ 50		37 (72.5)	53 (50.0)	
**Stage**	157			0.200**
I		12 (23.5)	17 (16.0)	
II		25 (49.0)	45 (42.5)	
III/IV		14 (27.5)	44 (41.5)	
**Tumor size**	157			0.979**
< 2cm		15 (29.4)	31 (29.2)	
2cm-5cm		28 (54.9)	57 (53.8)	
> 5cm or direct extension to chest wall		8 (15.7)	18 (17.0)	
**Grade**	145			< 0.001*
Low/Intermediate		15 (31.9)	75 (76.5)	
High		32 (68.1)	23 (23.5)	
**Mitotic count**	142			< 0.001*
≤14		26 (55.3)	81 (85.3)	
> 14		21 (44.7)	14 (14.7)	
**Nuclear pleomorphism**	142			< 0.001*
Low/Intermediate		10 (21.3)	62 (65.3)	
High		37 (78.7)	33 (34.7)	
**Tubule formation**	142			0.012*
≥10%		8 (17.0)	36 (37.9)	
< 10%		39 (83.0)	59 (62.1)	

[*] *P* value was calculated by using the Fisher's exact test.

[**] *P* value was calculated by using the Pearson's chi-squared test.
