# Supplementary material for: Molecular Characteristics and Metastasis Predictor Genes of Triple-Negative Breast Cancer: A Clinical Study of Triple-Negative Breast Carcinomas
Source: PLoS One. 2012 Sep 25;7(9):e45831. doi: 10.1371/journal.pone.0045831 (PMC3458056; doi:10.1371/journal.pone.0045831)
Supplement: Table S5 — Univariate and multivariate analyses for distant-metastasis-free survival were performed with each prognostic factor in our triple-negative patient dataset by using the Cox regression model. The multivariate analysis included 45 triple-negative breast cancer patients, owing to missing values in 3 patients. (PDF) [file pone.0045831.s008.pdf]

Prognostic factor	Univariate analysis		Multivariate analysis	
	Hazard ratio (95% CI)	*P* value	Hazard ratio (95% CI)	*P* value
**Stage:** III vs I/II	2.79 (0.75-10.39)	0.127	0.41 (0-105.71)	0.755
**Tumor size:** >5cm vs ≤5cm	2.08 (0.43-10.04)	0.361	0.05 (0-64.32)	0.416
**Grade:** high vs low/intermediate	0.75 (0.18-3.15)	0.696	0.14 (0-7.50)	0.330
**Lymph node metastasis:** positive vs negative	3.24 (0.81-13.00)	0.097	6.68 (0.01-472.00)	0.571
**Mitotic count:** >14 vs ≤14	1.31 (0.33-5.25)	0.704	1.10 (0-695.08)	0.977
**Nuclear pleomorphism:** low/intermediate vs high	1.17 (0.24-5.79)	0.851	6.56 (0.35-124.73)	0.211
**Tubule formation:** ≥10% vs <10%	1.61 (0.32-7.97)	0.562	0.56 (0-85.39)	0.822
**45-gene signature:** poor vs good prognosis group	78.82 (9.56-650.22)	< 0.001	623.18 (7.50-5176.00)	0.004
